# *Guazuma ulmifolia* Lam. Decreases Oxidative Stress in Blood Cells and Prevents Doxorubicin-Induced Cardiotoxicity

**DOI:** 10.1155/2018/2935051

**Published:** 2018-06-28

**Authors:** Jéssica Maurino dos Santos, Tamaeh Monteiro Alfredo, Katia Ávila Antunes, Janielle da Silva Melo da Cunha, Edna Márcia Almeida Costa, Emerson Silva Lima, Denise Brentan Silva, Carlos Alexandre Carollo, Wanderlei Onofre Schmitz, Ana Paula de Araújo Boleti, Edson Lucas dos Santos, Kely de Picoli Souza

**Affiliations:** ^1^Research Group on Biotechnology and Bioprospecting Applied to Metabolism (GEBBAM), Federal University of Grande Dourados, Dourados, MS, Brazil; ^2^Faculty of Pharmaceutical Sciences, Federal University of Amazonas, Manaus, AM, Brazil; ^3^Laboratory of Natural Products am Mass Spectrometry, Federal University of Mato Grosso do Sul, Campo Grande, MS, Brazil; ^4^University Hospital, Federal University of Grande Dourados, Dourados, MS, Brazil

## Abstract

Doxorubicin (DOX) is an efficient chemotherapeutic agent, but its clinical application is limited by its cardiotoxicity associated with increased oxidative stress. Thus, the combination of DOX and antioxidants has been encouraged. In this study, we evaluated (I) the chemical composition and antioxidant capacity of aqueous extracts from *Guazuma ulmifolia* stem bark (GUEsb) and leaves (GUEl) in 2,2-diphenyl-1-picrylhydrazyl (DPPH) free radical scavenging, 2,2′-azobis(2-amidinopropane) dihydrochloride- (AAPH-) or DOX-induced lipid peroxidation inhibition in human blood cells, and intracellular reactive oxygen species (ROS) quantification using the fluorescent probe dichloro-dihydro-fluorescein diacetate (DCFH-DA) in K562 erythroleukemia cells incubated with GUEsb and stimulated with hydrogen peroxide; (II) the viability of K562 cells and human leukocytes treated with GUEsb in the absence or presence of DOX using the 3-(4,5-dimethylthiazol-2-yl)-2,5-diphenyltetrazolium bromide (MTT) assay; (III) the acute toxicity of GUEsb; and (IV) the cardioprotective effect of GUEsb in C57Bl/6 mice treated with DOX. The chemical composition indicated the presence of flavan-3-ol derivatives and condensed tannins in GUEsb and glycosylated flavonoids in GUEl. GUEsb and GUEl showed free-radical scavenging antioxidant activity, antihemolytic activity, and AAPH- as well as DOX-induced malondialdehyde content reduction in human erythrocytes. Based on its higher antioxidant potential, GUEsb was selected and subsequently showed intracellular ROS reduction without impairing the chemotherapeutic activity of DOX in K562 cells or inducing leukocyte cell death, but protected them against DOX-induced cell death. Yet, GUEsb did not show *in vivo* acute toxicity, and it prevented MDA generation in the cardiac tissue of DOX-treated mice, thus demonstrating its cardioprotective effect. Taken together, the results show that GUEsb and GUEl are natural alternatives to treat diseases associated with oxidative stress and that, in particular, GUEsb may play an adjuvant role in DOX chemotherapy.

## 1. Introduction

Oxidative stress is a condition of imbalance between the quantity of reactive species and the inefficient activity of the antioxidant protection system of an organism [1], and it is frequently associated with symptoms and diseases, including diabetes [2], inflammation [3], gastrointestinal [4] and cardiovascular [5] diseases, and anthracycline-induced cardiotoxicity [6].

Doxorubicin (DOX), an anthracycline antibiotic, is widely used to treat solid and hematological cancers [7]. In cancer cells, DOX causes DNA intercalation and disrupts the cellular repair process, thus increasing the production of reactive oxygen species (ROS) and triggering oxidative stress [8]. Furthermore, studies indicate that DOX reduces the activity of the antioxidant enzymes superoxide dismutase (SOD) and catalase (CAT) in the heart [9, 10]. The resulting reactive oxygen species cause cumulative and irreversible cardiomyocyte damage that can lead to apoptosis or even to dysfunction as well as cardiac failure. Therefore, cardiotoxicity is the main limitation of its clinical application [11].

Studies have shown that DOX-induced cardiotoxicity can be reduced by the coadministration of DOX and extracts from medicinal plants with antioxidant activity, including *Camellia sinensis* [12] and *Capparis spinosa* [13], and by their combination with phenolic compounds [10, 14, 15].

*Guazuma ulmifolia* Lam. (Malvaceae), commonly known as “mutamba” [16] or “guácimo” [17], is found in Latin American countries, including Brazil [18]. In traditional medicine, it is used as an infusion or decoction to treat inflammation [19], gastrointestinal diseases [20], and diabetes [21], which are associated with oxidative stress [2–4]. Pharmacological studies have confirmed the antidiabetic potential of stem bark and leaves [22, 23], the hypotensive and vasorelaxant effects of *G. ulmifolia* stem bark [24], and the antihypercholesterolemic [25] and gastroprotective [26] activity of *G. ulmifolia* leaves. Phytochemical studies of *G. ulmifolia* leaves, fruits [21], and stem bark [27] identified phenolic compounds that are reported in the literature for their antioxidant activity [28–30] and that may contribute to the pharmacological activities described above.

In this context, we aimed to analyze the chemical composition and antioxidant capacity of aqueous extracts from *G. ulmifolia* stem bark and leaves in human blood cells subjected to different oxidative agents. Furthermore, we assessed the acute toxicity effects of *G. ulmifolia* stem bark extracts and their ability to prevent DOX-induced cardiotoxicity *in vivo*.

## 2. Materials and Methods

### 2.1. Botanical Material and Extract Preparation

*G. ulmifolia* stem bark and leaves were collected with the permission of the Brazilian Biodiversity Authorization and Information System (Sistema de Autorização and Informação sobre Biodiversidade, SISBIO; no. 51092), in the municipality of Ivinhema/Mato Grosso do Sul state (MS) 22° 22′ 22.08″ south, 53° 54′ 57.58″ west. The identification of the species was confirmed by a botany specialist, and a voucher specimen was deposited in the herbarium (DDMS) of the Federal University of Grande Dourados (UFGD), Dourados, MS, under record number 5815. After collection, the stem bark and leaves were washed in running water and dried in a convection oven at 40°C for 5 days and at 36°C for 7 days, respectively. Then, both samples were ground in a Willey knife mill, sieved through a 10 mm mesh, and stored in polypropylene containers at −20°C.

To prepare the aqueous extract from *G. ulmifolia* steam bark (GUEsb), 100 g of dried stem bark powder was decocted in 1 L of water for 15 min and cooled for 5 min. Subsequently, centrifugation was performed at 5000 rpm for 15 min, and the supernatant was freeze-dried and stored in a freezer at −20°C. The aqueous extract from *G. ulmifolia* leaves (GUEl) was prepared by infusing 100 g of dried leaf powder in 1 L of water heated to 80°C for 15 min, followed by cooling for 5 min. Then, the infusion was centrifuged at 5000 rpm for 15 min, and the supernatant was centrifuged for another 5 min, freeze-dried, and stored in a freezer at −20°C. The total yields were 22% for GUEsb and 7.4% for GUEl.

### 2.2. Chemical Composition

#### 2.2.1. Phytochemical Profile and Content

The phenolic content was determined using the method described by Meda et al. [31], with some modifications. Each extract was prepared at a final concentration of 100 *μ*g·mL^−1^ in 80% ethanol. A 0.5 mL aliquot of that solution was added to 2.5 mL of Folin–Ciocalteu reagent (1 : 10) and incubated at room temperature for 5 min. Subsequently, 2.0 mL of 14% sodium carbonate was added, followed by stirring and incubation in the dark for 2 h. A standard curve was constructed using aliquots of ethanolic solution of gallic acid (1 mg·mL^−1^) with different concentrations (0.4–21.0 *μ*g·mL^−1^). The absorbance was read at 760 nm against an 80% ethanol blank in a spectrophotometer (T70 UV/VIS Spectrometer, PG Instruments Ltd). The equation of the curve was derived by linear regression correlation between the gallic acid concentration and each absorbance reading, thus indirectly calculating the total phenolic content of each extract. Each sample was tested in triplicate, resulting in a mean value expressed as milligram equivalents of gallic acid per gram of extract (mg EGA·g^−1^ extract).

The total flavonoid contents of GUEsb and GUEl were determined as described by Liberio et al. [32], with some modifications. For such a purpose, each extract was prepared at a final concentration of 100 *μ*g·mL^−1^ in methanol PA. A 0.5 mL aliquot of that solution was added to 4.5 mL of aluminum chloride (2%) and incubated at room temperature for 30 min. A standard curve was constructed using aliquots of the methanolic solution of quercetin (1 mg·mL^−1^) with different concentrations (0.4–21.0 *μ*g·mL^−1^). The absorbance was read at 415 nm against a methanol blank. The equation of the curve was derived by linear regression correlation between the quercetin concentration and each absorbance reading, thus indirectly calculating the total flavonoid content of each extract. Each sample was tested in triplicate, resulting in a mean value expressed as milligram equivalents of quercetin per gram of extract (mg EQ·g^−1^ extract).

### 2.3. Antioxidant Potential

#### 2.3.1. DPPH Free Radical Scavenging

The 2,2-diphenyl-1-picrylhydrazyl (DPPH, Sigma-Aldrich) free radical scavenging activities of GUEsb and GUEl were assessed as described by Gupta and Gupta [33] with some modifications. A total of 200 *μ*L of GUEsb or GUEl at different concentrations (1–2000 *μ*g·mL^−1^) was added to 1800 *μ*L of DPPH solution (0.11 mM) in 80% ethanol. The mixture was homogenized, incubated for 30 min at room temperature in the dark, and then read in a spectrophotometer at 517 nm against an 80% ethanol blank. Ascorbic acid (AA) and butylated hydroxytoluene (BHT) were used as standard antioxidants. Three independent experiments were performed in triplicate for each extract. The data were expressed as the concentration necessary to inhibit 50% of the free radical (IC_50_) and as the maximum activity (*A*_max_). The percentage of inhibition in relation to the control (DPPH solution (0.11 mM)) was calculated using the following equation:

(1) $$\begin{array}{c} \%DPPH\;inhibition=\left(\frac{{Abs}_{control}-{Abs}_{sample}}{{Abs}_{control}}\right)\times 100. \end{array}$$

#### 2.3.2. Preparation of the Human Erythrocyte Suspension (10%)

After approval of the study by the UFGD Research Ethics Committee under protocol number 073238/2016, peripheral blood samples (10 mL) were collected from healthy donors in tubes with sodium citrate and centrifuged at 2000 rpm for 5 min. Then, the plasma and leukocytes were removed, and the erythrocytes were subjected to three washes with saline (0.9% NaCl) at 2000 rpm, discarding the supernatant after each washing cycle. Subsequently, a solution of erythrocytes (10%) was prepared in 0.9% NaCl.

#### 2.3.3. Hemolytic Activity of *G. ulmifolia* Extracts

The human erythrocyte suspension (10%) was incubated at 37°C for 30 min with different concentrations (25, 50, 100, 250, 500, and 1000 *μ*g·mL^−1^) of GUEsb, GUEl, or AA (antioxidant standard). Then, 0.5 mL of 0.9% NaCl was added. After 240 min, the samples were centrifuged at 2000 rpm for 5 min, and the absorbance was read at 540 nm. Erythrocytes incubated with only 0.9% NaCl were used as controls [34].

#### 2.3.4. Oxidative Hemolysis Inhibition in Human Erythrocytes Induced by 2,2′-Azobis(2-Amidinopropane) Dihydrochloride (AAPH) or DOX

The ability of GUEsb and GUEl to decrease AAPH-induced oxidative stress in human erythrocytes was assessed following the method described by Campos et al. [34] with some modifications. For such a purpose, the erythrocyte suspension was preincubated at 37°C for 30 min with different concentrations (25, 50, 100, 250, 500, and 1000 *μ*g·mL^−1^) of GUEsb, GUEl, or AA (antioxidant standard). Then, 0.5 mL of AAPH (50 mM diluted in 0.9% NaCl) or DOX (300 *μ*g·mL^−1^ diluted in 0.9% NaCl) solution was added. After 240 min, the samples were centrifuged at 2000 rpm for 10 min and read in a spectrophotometer at 540 nm. Total hemolysis was induced by incubation of the erythrocyte suspension in distilled water. Erythrocytes incubated with only AAPH or DOX were used as controls. Three independent experiments were conducted in duplicate for each extract. The percentage of hemolysis was calculated using the following formula:

(2) $$\begin{array}{c} Hemolysis\;\left(\%\right)=\left({Abs}_{sample}÷{Abs}_{total\;hemolysis}\right)\times 100. \end{array}$$

#### 2.3.5. Malondialdehyde (MDA) Dosage

After 240 min of erythrocyte suspension incubation with the extract and the oxidative hemolysis inducer (AAPH or DOX), the samples were centrifuged, and a 0.5 mL aliquot of supernatant was added to a tube with 1 mL of 10 nM thiobarbituric acid (TBA, Merck, diluted in 75 mM monobasic potassium phosphate buffer, pH = 2.5), which was incubated in a water bath at 96°C for 45 min. Then, the samples were cooled in an ice bath for 15 min. Subsequently, each sample was added to 4 mL of butanol, homogenized, and centrifuged at 3000 rpm for 5 min, and the absorbance was read at 532 nm [34]. A total of 0.5 mL of 20 mM MDA and 1 mL of TBA solution was used as a control. Three independent experiments were performed in duplicate for each extract. The MDA content was expressed using the following formula:

(3) $$\begin{array}{c} MDA\;\left(nmol\cdot {mL}^{-1}\right)={Abs}_{sample}\times \left(\frac{20\times 220.32}{{Abs}_{control}}\right). \end{array}$$

### 2.4. Cell Culture

#### 2.4.1. Cell Culture Conditions

In this study, we used the chronic myeloid leukemia (K562) cell line cultured in RPMI 1640 media (Gibco, Brazil) supplemented with 10% fetal bovine serum (FBS), 100 U·mL^−1^ penicillin, and 100 *μ*g·mL^−1^ streptomycin (Gibco, Brazil) at 37°C in an incubator with 5% CO_2_.

#### 2.4.2. Cellular Antioxidant Activity

GUEsb was selected for the other studies because it showed the best overall antioxidant activity. To assess the intracellular ROS scavenging capacity of GUEsb, we used the probe 2′,7′-dichlorofluorescin diacetate (DCFH-DA), according to the method by Wolfe and Liu [35] with some modifications. K562 cells (2 × l0^4^ cells well^−1^ in 96-well microplates) were incubated at 37°C with 20 *μ*M DCFH-DA for 1 h, washed in Hank's balanced salt solution, and treated with different concentrations of GUEsb (3.12, 6.25, 12.5, and 25 *μ*g·mL^−1^) as well as 500 *μ*M H_2_O_2_. The fluorescence was measured for 1 h every 5 min at an excitation wavelength of 485 nm and at an emission wavelength of 520 nm using a microplate reader (DTX 800, Beckman, CA, USA). Cells with and without H_2_O_2_ in the presence of DCFH-DA were used as positive and negative controls, respectively. Quercetin was used as the antioxidant standard. Two independent experiments were performed in triplicate. The intracellular antioxidant activity was expressed as the percentage of inhibition of intracellular ROS produced by exposure to H_2_O_2_.

(4) $$\begin{array}{c} Intracellular\;ROS\;level\;\left(\%\right)=\left({Abs}_{sample}÷{Abs}_{Positive\;control}\right)\times 100. \end{array}$$

#### 2.4.3. Cell Viability Assay

We assessed whether GUEsb affects the cytotoxic activity of DOX in K562 cells and whether it is able to decrease or inhibit DOX-induced human leukocyte death, according to the method by Mosmann [36], with some adaptations. The IC_50_ of DOX (0.5 *μ*g·mL^−1^) in K562 cells was previously determined. To isolate leukocytes, total blood was diluted in 0.9% NaCl, transferred into a sterile tube with Ficoll–Paque at a 3 : 1 ratio, and centrifuged at 2000 rpm for 20 min. Then, the plasma was discarded, and the layer of leukocytes was washed 2x in 0.9% NaCl. After the preparation procedures, K562 cells (2 × l0^4^ cells well^−1^) or leukocytes (12 × 10^4^ cells well^−1^) were plated in 96-well microplates and treated with 50 *μ*L of different concentrations of GUEsb (1.56, 3.12, 6.25, 12.5, and 25 *μ*g·mL^−1^) in the presence or absence of 50 *μ*L of DOX at its IC_50_ value (0.5 *μ*g·mL^−1^, diluted in 0.9% NaCl) for 24, 48, and 72 h. DOX and culture medium were used as positive and negative controls, respectively. After the incubation period, the cells were centrifuged at 1500 rpm for 10 min and washed in phosphate-buffered saline (PBS), followed by the subsequent addition of 100 *μ*L of 3-(4,5-dimethylthiazol-2-yl)-2,5-diphenyltetrazolium bromide (MTT) solution (1 mg·mL^−1^ diluted in culture medium). After 240 min of incubation, the formazan crystals were resuspended in 100 *μ*L of dimethylsulfoxide (DMSO), and the sample absorbance was read at 630 nm in a Thermoplate TP-READER. Three independent experiments were performed in triplicate. The cell viability was calculated using the following formula:

(5) $$\begin{array}{c} Cell\;viability\;\left(\%\right)=\left({Abs}_{sample}÷{Abs}_{Negative\;control}\right)\times 100. \end{array}$$

### 2.5. Animals

#### 2.5.1. Animal Maintenance

This study was approved by the UFGD Ethics Committee on Animal Use, protocol number 29/2016, and was conducted in accordance with the ethical principles of animal experimentation adopted by the National Council for the Control of Animal Experimentation (Conselho Nacional de Controle de Experimentação Animal (CONCEA)). The animals were maintained under controlled temperature (22 ± 2°C) conditions and a 12 h light–dark cycle, and they were fed ad libitum.

#### 2.5.2. Acute Toxicity Test in C57Bl/6 Mice

Acute toxicity was tested based on protocols from the Organization for Economic Cooperation and Development (OECD) Guideline 425 [37]. On the 1st day, one female C57Bl/6 mouse received 2000 mg·kg^−1^ of GUEsb orally (p.o.) after fasting for 8 h. The animal was regularly observed in the first 24 h. Subsequently, four other animals were subjected to the same procedure. The experimental procedure was repeated at a dose of 5000 mg·kg^−1^ towards defining the median lethal dose (LD_50_) for the animals. Control animals (*n* = 5) received only water (orally). Then, the animals were observed once daily for 14 days. The body mass along with the food and water intake were recorded regularly. Hippocratic screening was performed to assess physiological and behavioral parameters (defecation, urination, exophthalmos, piloerection, tremors, hypersalivation, catatonia, tail erection, lacrimation, ataxia, pallor/hyperemia/cyanosis of the ears, paw licking, nose scratching, and tail biting). At the end of the study period, all animals were subjected to anesthesia with ketamine/xylazine and then euthanized. The organs (central nervous system, heart, liver, spleen, lungs, and kidneys) were removed, weighed, and macroscopically analyzed. Blood was drawn for biochemical and hematological analysis.

#### 2.5.3. DOX-Induced Cardiotoxicity in C57Bl/6 Mice

*In vivo* cardiotoxicity was induced by DOX, according to Momin et al. [9], with some modifications. Male C57Bl/6 mice of approximately 25 g were randomly distributed between groups (*n* = 5). The groups were treated as follows: (I) control (water, p.o.), (II) DOX (water, p.o.), and (III) DOX + GUEsb (200 mg GUEsb·kg^−1^ body mass, p.o.). From the 7th day, the animals received, in combination with GUEsb, the cumulative dose of DOX (totaling 24 mg·kg^−1^ diluted in 0.9% NaCl) by intraperitoneal injection (i.p.)) divided into six doses on alternate days (7th, 9th, 11th, 13th, 15th, and 17th). On the 18th day, the animals were anesthetized with ketamine/xylazine and euthanized, and the organs (heart, liver, and kidneys) were collected, weighed, and macroscopically evaluated. Furthermore, the following parameters were assessed: changes in body mass, food and water intake, relative organ mass, and MDA content of the heart.

*(1) MDA Dosage*. The MDA content of the heart was assessed according to the method adapted from Draper et al. [38]. The heart was triturated in 1.15% potassium chloride (KCl) and centrifuged at 3000 rpm for 10 min. Then, 0.5 mL of the supernatant was incubated with 1 mL of 10% trichloroacetic acid (TCA) and 1 mL of 20 nM TBA (diluted in 75 mM monobasic potassium phosphate buffer, pH = 2.5) at 96°C for 45 min. After cooling, 3 mL of butanol was added. The mixture was homogenized and centrifuged at 3000 rpm for 5 min, and the absorbance was read at 532 nm. The control solution was 0.5 mL of 20 mM MDA and 1 mL of TBA. The MDA content was expressed using the following formula:

(6) $$\begin{array}{c} MDA\;\left(nmol\cdot {mL}^{-1}\right)={Abs}_{sample}\times \left(\frac{20\times 220.32}{{Abs}_{control}}\right). \end{array}$$

### 2.6. Statistical Analysis

The results were expressed as the mean ± standard error of the mean (SEM). The results were compared by analysis of variance (ANOVA) followed by the Student–Newman–Keuls posttest. Data were considered significant when *P* < 0.05. Statistical tests were performed using the statistical software GraphPad Prism 5.0.

## 3. Results

### 3.1. Chemical Composition

**Figure 1:**
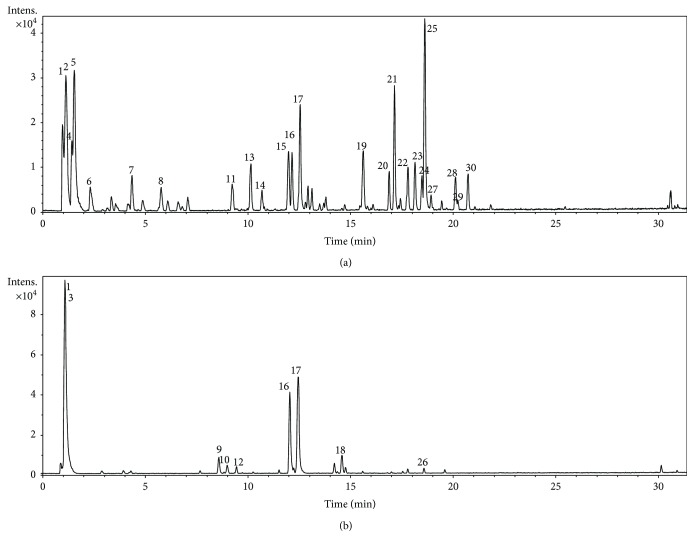
Total ion chromatogram in negative ion mode of aqueous extract from leaves (a) and stem bark (b) of *Guazuma ulmifolia*.

The chemical profile of *G. ulmifolia* extracts was identified based on UV, precise mass and tandem mass spectrometry (MS/MS) data compared with published data and the coinjection of standards (Figure 1). Compounds relative to the thirty chromatographic peaks were detected in the aqueous extracts of *G. ulmifolia* leaves and stem bark, and the main compounds identified were flavan-3-ol-derived flavonoids, including monomers and dimers, condensed tannins in GUEsb, and glycosylated flavonoids in GUEl (Table 1). The phenolic and flavonoid contents were 324.4 ± 4.1 and 240.0 ± 0.4 mg GAE·g^−1^ extract along with 12.9 ± 1.0 and 32.5 ± 1.3 mg EQ·g^−1^ extract in GUEsb and GUEl, respectively.

### 3.2. Antioxidant Capacity Assessment and Decreased Oxidative Stress

#### 3.2.1. DPPH Free Radical Scavenging

GUEsb and GUEl showed high DPPH free radical scavenging activities, similar to that of the lipophilic antioxidant control BHT and lower than that of the hydrophilic antioxidant control AA, as shown by the IC_50_ and *A*_max_ values outlined in Table 2.

#### 3.2.2. Determination of the Hemolytic Activity, AAPH-Induced Oxidative Hemolysis Inhibition, and MDA Dosage

**Figure 2:**
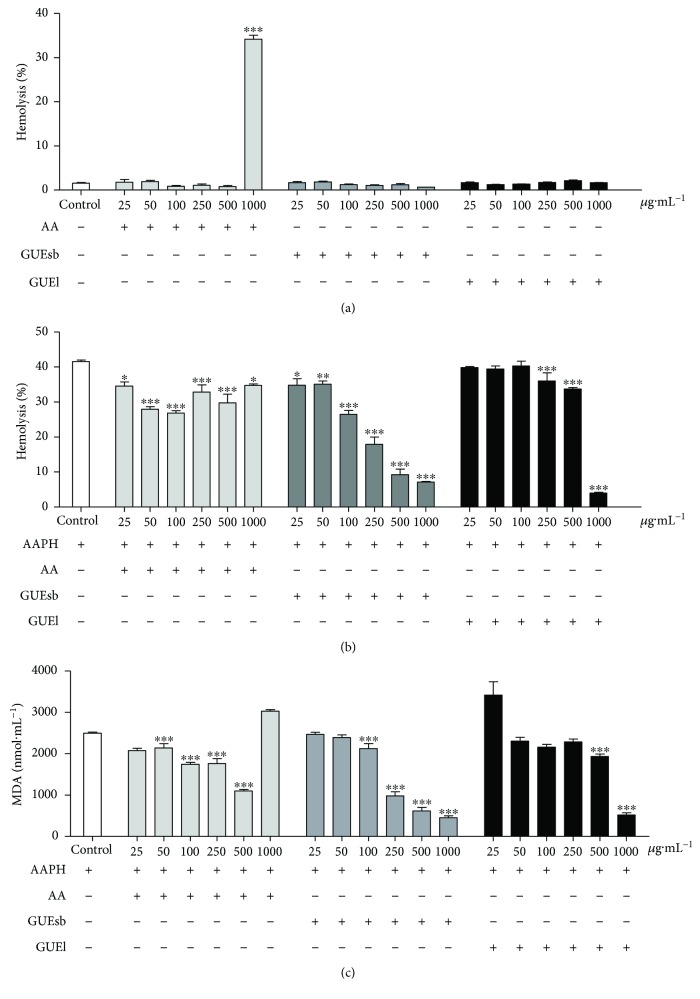
Hemolysis and MDA content of human erythrocytes. AA, GUEsb, or GUEl activity on (a) hemolysis, (b) hemolysis inhibition, and (c) malondialdehyde (MDA) content resulting from AAPH-induced lipid peroxidation. The data are expressed as the mean ± SEM. ^∗^*P* < 0.05, ^∗∗^*P* < 0.01, and ^∗∗∗^*P* < 0.001 compared with the control (erythrocytes incubated with only AAPH). AA = ascorbic acid; GUEsb = aqueous extract from *G. ulmifolia* stem bark; GUEl = aqueous extract from *G. ulmifolia* leaves.

GUEsb and GUEl showed no hemolytic activity at the concentrations tested, which was observed only at the highest concentration of ascorbic acid (Figure 2(a)). Then, the antioxidant potentials of GUEsb and GUEl against AAPH-induced hemolysis were analyzed. Both extracts decreased AAPH-induced hemolysis at 240 min of incubation more efficiently than AA; 25 and 1000 *μ*g mL^−1^ GUEsb induced 16% and 83% protection, respectively, and GUEl induced 13% and 90% protection at 250 and 1000 *μ*g mL^−1^, respectively (Figure 2(b)).

Subsequent tests showed that both extracts decreased lipid peroxidation, as indicated by MDA levels lower than those of the control group (Figure 2(c)). GUEsb decreased MDA by 15% and 82% at 100 and 1000 *μ*g mL^−1^, and GUEl decreased MDA by 14% and 79% at 500 and 1000 *μ*g mL^−1^, respectively. Comparatively, AA decreased MDA production by 14% and 56% at 50 *μ*g·mL^−1^ and 500 *μ*g·mL^−1^, respectively, and AA showed oxidant activity at the highest concentration tested (Figure 2(c)).

#### 3.2.3. Inhibition of DOX-Induced Oxidative Hemolysis and MDA Production

**Figure 3:**
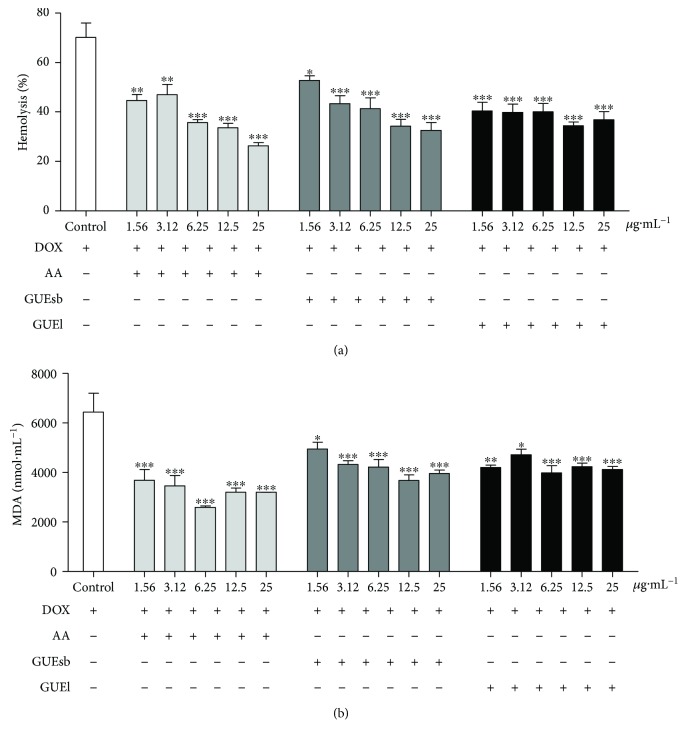
DOX-induced hemolysis and MDA content of human erythrocytes incubated for 240 min with AA, GUEsb, or GUEl (1.56–25 *μ*g·mL^−1^). (a) Hemolysis inhibition at 240 min after adding DOX [300 *μ*g·mL^**−1**^]. (b) Malondialdehyde (MDA) content resulting from DOX-induced lipid peroxidation [300 *μ*g·mL^**−1**^] after 240 min. The data are expressed as the mean ± SEM. ^∗^*P* < 0.05, ^∗∗^*P* < 0.01, and ^∗∗∗^*P* < 0.001 compared with the control (erythrocytes incubated with DOX only). AA = ascorbic acid; GUEsb = aqueous extract from *G. ulmifolia* stem bark; GUEl = aqueous extract from *G. ulmifolia* leaves.

When testing for protection against DOX-induced hemolysis, GUEsb and GUEl were able to protect human erythrocytes against oxidative hemolysis (Figure 3(a)) and MDA production (Figure 2(b)) after 240 min of incubation at all of the concentrations tested. The highest degrees of protection against hemolysis for GUEsb and GUEl were 54% and 48% at 25 *μ*g·mL^−1^, respectively. This protection was similar to that of the antioxidant standard AA, which was 62% at the same concentration (Figure 2(a)).

DOX-induced MDA production was also decreased by 38% and 36% upon incubation with 25 *μ*g·mL^−1^ GUEsb and GUEl, respectively, compared with a 50% decrease caused by AA at the same concentration (Figure 3(b)).

#### 3.2.4. Cellular Antioxidant Activity

**Figure 4:**
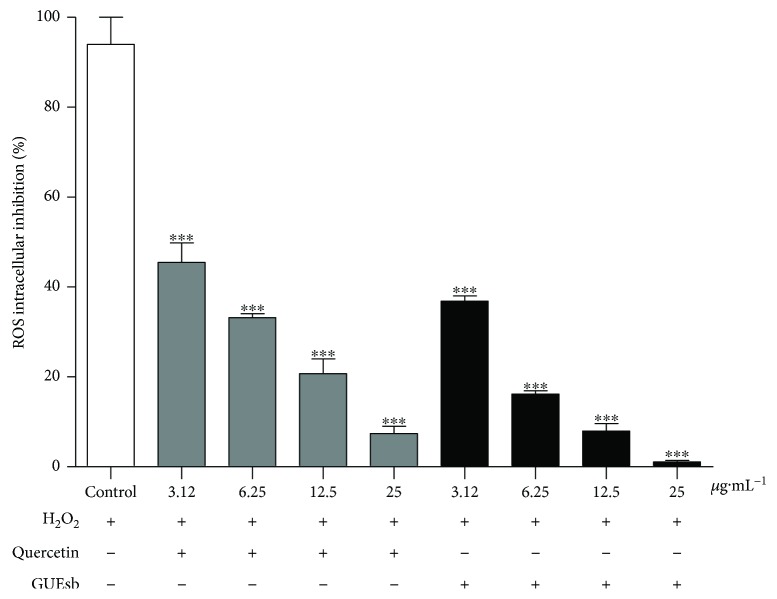
Cellular antioxidant activity. Inhibition of intracellular ROS production in K562 erythroleukemia cells incubated with DCFH-DA for 1 h, subsequently treated with quercetin or GUEsb (3.12, 6.25, 12.5, and 25 *μ*g·mL^−1^) and immediately exposed to hydrogen peroxide (H_2_O_2_ 500 *μ*M). The data are expressed as the mean ± SEM. ^∗∗∗^*P* < 0.001 compared with the control (cells incubated with DCF and exposed to H_2_O_2_). GUEsb = aqueous extract from *G. ulmifolia* stem bark.

We continued the studies only with GUEsb because it showed a higher overall antioxidant potential. K562 erythroleukemia cells subjected to H_2_O_2_-induced oxidative stress showed high intracellular ROS production, which was decreased by incubation with GUEsb at all of the concentrations tested, similarly to the activity of the antioxidant standard quercetin (Figure 4).

### 3.3. Cell Viability

#### 3.3.1. Viability of K562 Erythroleukemia Cells Treated with GUEsb and Incubated with or without DOX

**Figure 5:**
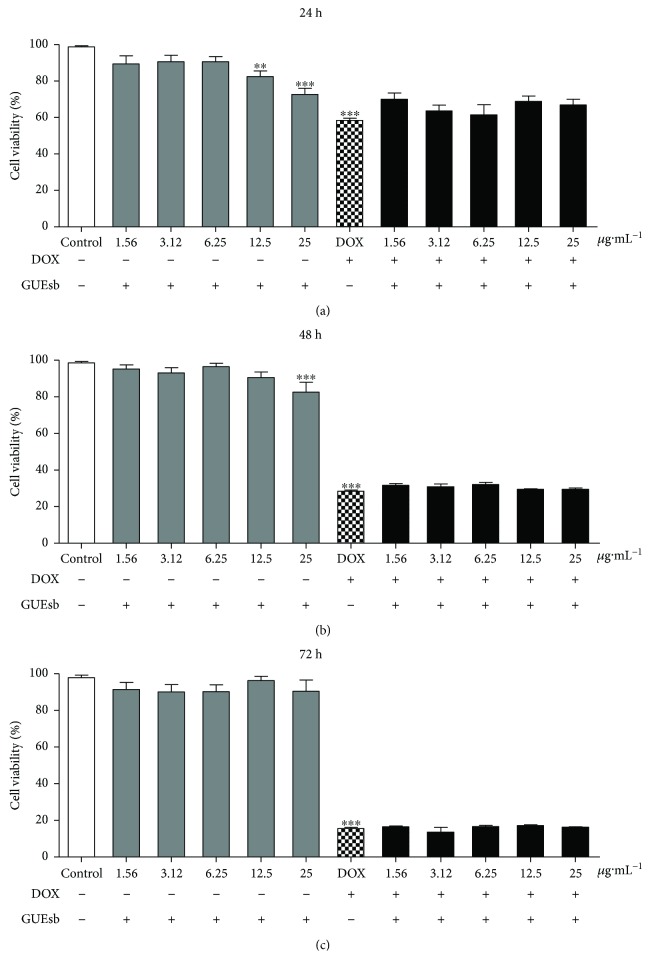
DOX-induced cytotoxicity in K562 erythroleukemia cells treated with GUEsb (1.56–25 *μ*g·mL^−1^) for 24, 48, and 72 h. Viability of K562 cells treated with GUEsb and incubated with or without DOX (0.5 *μ*g·mL^−1^) for (a) 24, (b) 48, and (c) 72 h. The data are expressed as the mean ± SEM. Only the cells treated with GUEsb were compared with the control (K562 cells incubated with culture media only), and significant differences were identified when ^∗∗^*P* < 0.01 and ^∗∗∗^*P* < 0.001. The cells treated with DOX + GUEsb were compared with DOX (K562 cells incubated with 0.5 *μ*g·mL^−1^ DOX). GUEsb = aqueous extract from *G. ulmifolia* stem bark.

K562 cells incubated only with GUEsb showed decreased cell viability by 18% and 27% at 12.5 and 25 *μ*g·mL^−1^, respectively, at 24 h of incubation and by 18% at 25 *μ*g·mL^−1^ and 48 h of incubation (Figure 5). K562 cells incubated with 0.5 *μ*g·mL^−1^ DOX (the DOX IC_50_ of that cell line was previously determined) showed 42%, 72%, and 84% cell death at 24, 48, and 72 h of incubation, respectively. Combined treatment with DOX + GUEsb caused no change in the DOX-induced cell death profile, leading to similar cell death rates of 33%, 71%, and 84%, at the same incubation times, respectively.

#### 3.3.2. Viability of Human Leukocytes Treated with GUEsb and Incubated with or without DOX

**Figure 6:**
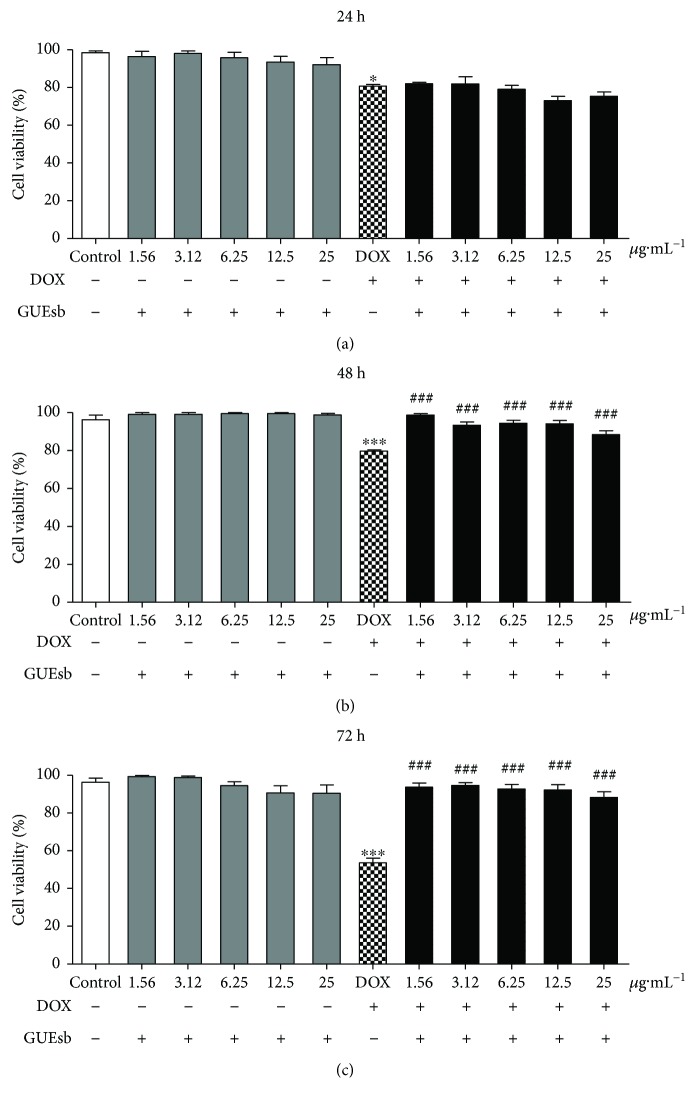
DOX-induced cytotoxicity in human leukocytes treated with GUEsb (1.56–25 *μ*g·mL^−1^) for 24, 48, and 72 h. Viability of human leukocytes treated with GUEsb and incubated with or without DOX (0.5 *μ*g·mL^−1^) for (a) 24, (b) 48, and (c) 72 h. The data are expressed as the mean ± SEM. Only the cells treated with GUEsb were compared with the control (human leukocytes incubated with culture media only), and significant differences were identified when ^∗^*P* < 0.05 and ^∗∗∗^*P* < 0.001. The cells treated with DOX + GUEsb were compared with DOX (human leukocytes incubated with 0.5 *μ*g·mL^−1^ DOX), and significant differences were identified when ^###^*P* < 0.001. GUEsb = aqueous extract from *G. ulmifolia* stem bark.

Leukocytes treated with only GUEsb showed no decrease in cell viability at any of the concentrations and times tested. However, leukocytes incubated with 0.5 *μ*g·mL^−1^ DOX showed 19%, 20%, and 46% cell death after 24, 48, and 72 h of incubation, respectively. The combined treatment with 25 *μ*g·mL^−1^ DOX + GUEsb was able to prevent DOX-induced cell death by 9% and 35% at 48 and 72 h, respectively (Figure 6).

### 3.4. Animals

#### 3.4.1. Acute Toxicity Test in C57Bl/6 Mice

Female C57Bl/6 mice treated with 2000 and 5000 mg GUEsb·kg^−1^ body mass showed no signs of toxicity (Table 3), mortality, or physical and behavioral changes, except for an increase in creatinine at the highest dose, compared with the control group.

#### 3.4.2. DOX-Induced Cardiotoxicity in C57Bl/6 Mice

*(1) Body Mass, Food Intake, and Relative Organ Mass*. Mice treated with DOX showed decreased body mass at the end of the treatment compared with the control group (Table 4). No changes were observed in the other parameters.

**Figure 7:**
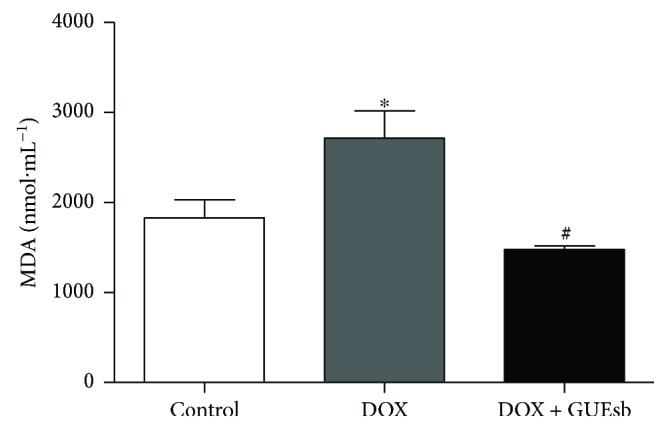
DOX-induced cardiotoxicity in C57Bl/6 mice. MDA content of the control (water), DOX (water + DOX cumulative dose of 24 mg·kg^−1^), and DOX + GUEsb (GUEsb 200 mg·kg^−1^ + DOX cumulative dose of 24 mg·kg^−1^) mouse heart tissue after 18 days. The data are expressed as the mean ± SEM (*n* = 5). ^∗^*P* < 0.05 compared with control and ^#^*P* < 0.05 compared with DOX. GUEsb = aqueous extract from *G. ulmifolia* stem bark.

*(2) Inhibition of DOX-Induced MDA Content in the Cardiac Tissue*. Treatment with DOX increased the cardiac MDA content by approximately 48% compared with the control group. Combined treatment with DOX and GUEsb prevented this MDA production in the cardiac tissue and reduced the cardiac MDA content in the animals of the DOX + GUEsb group by 19% compared with the control group (Figure 7).

## 4. Discussion

Medicinal plants are key targets in the search for therapeutic alternatives against oxidative stress because some phytochemicals, such as phenolic compounds, have antioxidant properties capable of maintaining the redox balance and protecting cells against damage caused by excess ROS [39]. In this study, several compounds, previously described in the literature, were identified in *G. ulmifolia* stem bark, such as phenolic acids, flavan-3-ol-derived flavonoids (monomers and dimers), and condensed tannins, including epicatechin, epigallocatechin, catechin, procyanidins, prodelphinidin–procyanidin, and procyanidin–profisetinidin [40, 41]. Phenolic acids and glycosylated flavonoids (with one, two, or three sugars), including chlorogenic acid, catechin, quercetin, and luteolin, were identified in leaf extracts [18, 42]. Furthermore, unpublished compounds were identified, namely, citric and quinic acids in *G. ulmifolia* stem bark and *O*-pentosyl quercetin, di-*O*-deoxyhesosyl-hesosyl quercetin, *O*-deoxyhexosyl hexosyl luteolin, and di-O-deoxyhexosyl-hexosyl kaempferol in *G. ulmifolia* leaves.

The quantity of phenolic compounds can directly affect the biological potential of natural products [43], including the antioxidant activity of medicinal plants [43–45]. In this study, a high phenolic content was found in both extracts, and GUEsb showed a higher phenolic content than GUEl and one similar to that found by Feltrin et al. [27] in 70% hydroethanolic extract from *G. ulmifolia* stem bark. GUEsb showed a higher DPPH radical scavenging activity than GUEl. The highest flavonoid content was found in GUEl, which was even higher than that found by Morais et al. [42] in the ethanolic extract from *G. ulmifolia* leaves, and our GUEl showed a higher free-radical scavenging capacity than that found in the previous study. When compared with antioxidant standards, both extracts were inferior to AA and similar to the antioxidant standard BHT, an isolated synthetic compound widely used in the cosmetic, pharmaceutical, and food industries [46], which has been associated with the development of cardiac diseases and carcinogenesis [47, 48], thus indicating the need for new substitutes, particularly natural compounds. Taken together, this evidence supports the traditional medicine [19] procedure of aqueous extraction as an efficient method to isolate bioactive compounds present in *G. ulmifolia*.

To best understand the biological potential of the *G. ulmifolia* extracts, we used human blood cells subjected to oxidative stress induced by different oxidant agents. Initially, we used AAPH, a water-soluble azo compound that decomposes at 37°C generating peroxyl radicals (ROO) [49] responsible for oxidizing erythrocyte membrane lipids and proteins [50]. Azo compound-derived ROO and those formed physiologically and pathologically *in vivo* react with biomolecules similarly, facilitating the study of the oxidation kinetics of biological molecules and their possible protection [51]. Both extracts, GUEsb and GUEl, decreased human erythrocyte lysis and the content of MDA produced, even more efficiently than the antioxidant standard AA, which has a lower protective activity and even behaved as an oxidant at the highest concentration tested, which may be related to Fenton's reaction. In this process, ascorbate reduces metal ions, thereby generating intermediate radicals [52, 53]. The protective effect of GUEsb against oxidative hemolysis was even stronger than those of other extracts in the same biological model [43, 54].

Erythrocytes were also exposed to another oxidant agent, the chemotherapeutic doxorubicin, which is widely used to treat several types of cancer. However, the oxidative stress generated by this drug is indicated as one of the main inducers of cardiotoxicity leading to the development of severe heart diseases [11]. Approximately 30% of patients subjected to chemotherapy with DOX develop cardiac dysfunction [55]. In this context, efforts have been directed towards searching for antioxidant compounds, such as dexrazoxane, which are able to prevent or attenuate the toxicity caused by this drug, and this topic is one of the focuses of discussion of the International Cardioncology Society [56].

In this study, the oxidative stress signs in human erythrocytes exposed to DOX, including increased hemolysis and MDA, were reduced by the combined use of GUEsb or GUEl with DOX. The antioxidant activity of *G. ulmifolia* extracts against AAPH- and DOX-induced oxidative stress may be partly attributed to the presence of phenolic compounds because they are able to chelate metal ions and inhibit Fenton's reaction, particularly flavonoids such as quercetin present in leaves and catechin present in the stem bark [57]. Moreover, the presence of aromatic rings allows H^+^ and electron donation, preventing the formation of ROS, such as OH^+^ and ROO [58], which explains the decrease in lipid peroxidation.

The phytochemical composition and the previous results indicated a higher antioxidant potential of GUEsb, which was selected for the other tests. Subsequently, we confirmed, using a fluorescent probe, that GUEsb induced intracellular ROS scavenging in a K562 erythroleukemia line exposed to the oxidant agent H_2_O_2_ as efficiently as the control quercetin. This detoxification role may be played by both catechin [59] and quinic acid [60] or even by the synergism between them, resulting in increased CAT activity, which is the enzyme responsible for converting H_2_O_2_ into water molecules.

Antioxidants can attenuate oxidative damage and become promising strategies in chemotherapy, but the anticancer activity of the drug must not be impaired [61]. Although GUEsb caused a slight increase in cell death at the initial treatment times, when combined with DOX in K562 erythroleukemia cells, it had no effect on DOX-induced cell death. The ability to attenuate oxidative stress without affecting the cytotoxic activity of DOX is a key characteristic for the application of GUEsb as an adjuvant and may be related to the presence of flavonoids, which can reduce the negative effects of DOX without affecting the activity of the drug [62].

In addition to oxidative stress, DOX impairs leukocyte formation, causing leucopenia [63], most likely linked to the high content of polyunsaturated fatty acids in the membrane of those cells, which renders them highly sensitive to ROS [64]. GUEsb has immunoprotective effects on this condition, preventing DOX-induced death. This activity may be related to the antioxidant properties of the phenolic compounds of GUEsb. Furthermore, it should be noted that GUEsb contains procyanidins, which are associated with improved leucopenia symptoms in animals subjected to chemotherapy-induced immunosuppression [65].

However, cardiotoxicity is still the major limitation for the clinical application of DOX [11, 62]. The mechanism of anthracycline-induced cardiotoxicity is unclear, although the most commonly discussed hypotheses are DNA damage by increased production of reactive species and mitochondrial dysfunction caused by inhibition of topoisomerases II, which are the mechanisms of action of DOX in cancer cells [8]. Some factors increase the heart susceptibility to DOX-induced toxicity, such as high oxidative metabolism, decreased antioxidant enzymes [66], and, especially, the high DOX affinity for cardiolipin, a phospholipid essential to the mitochondrial structure and function as well as the energy metabolism of cardiomyocytes [67]. The formation of a strong DOX–cardiolipin complex results in DOX retention within the mitochondrial membrane, allowing continuous redox cycles, thereby causing oxidative damage [6]. However, inhibition of topoisomerases II is indicated as the main mediator of DOX-induced cardiotoxicity, since this drug promotes intercalation into the base pairs and topoisomerase-II*α* inhibition-mediated disruption of DNA repair and mitochondrial dysfunction as a consequence topoisomerase-II*β* inhibition-mediated peroxisome proliferator-activated receptor (PPAR) suppression, leading to cell death [8, 68, 69]. Consequently, both mechanisms culminate in the leads to the loss of functional myocytes and to irreversible cardiac tissue damage because these cells do not regenerate [68].

DOX coadministration with natural antioxidants, including isolated phenolic compounds [10, 14, 15], and extracts from medicinal plants, such as *Ixora coccinea* Linn [9], *Camellia sinensis* [12], *Capparis spinosa* [13], *Vaccinium macrocarpon* [70], and *Melissa officinalis* [71], aims at finding alternative therapies to mitigate cardiac damage. In this study, GUEsb-induced cardioprotection in animals treated with DOX was stronger than that of other plants, which, even at higher doses [12, 71], only mitigated DOX-induced cardiotoxicity in rats. GUEsb was able to prevent MDA production in the cardiac tissue of animals treated with DOX. Previous studies indicate that procyanidin [72] and catechin [59], compounds also found in GUEsb, are able to reduce DOX-induced lipid peroxidation. Moreover, catechins have chelating properties and modulate the activity of antioxidant enzymes (SOD, CAT, and glutathione peroxidase) [59]. Accordingly, GUEsb may have been able to prevent DOX complexation with iron ions and to enhance ROS detoxification in the cardiac tissue. Our results suggest that the cardioprotective effects of GUEsb result from oxidative stress suppression mediated by its phytochemical constituents, which was corroborated by direct ROS scavenging and decreased lipid peroxidation in human erythrocytes and mice cardiomyocytes.

Toxicity data indicate that GUEsb is safe for consumption, based on acute lethality tests, physical and behavioral changes, and biochemical and hematological parameters assessing the toxic effects of several plant extracts in animal models [73–76]. GUEsb induced no physical or behavioral changes in the animals tested nor any changes in the food and water intake, body mass, relative organ mass, or biochemical and hematological parameters. Therefore, GUEsb is safe for consumption.

## 5. Conclusion

Taken together, our results show that GUEsb and GUEl have antioxidant activity and are able to decrease oxidative stress in human blood cells, including DOX-induced oxidative stress, indicating that both extracts are possible, natural alternatives to treat diseases associated with oxidative stress. Furthermore, GUEsb showed no effect on the cytotoxicity of the drug or toxicity and was able to suppress DOX-induced cardiotoxicity.

## Figures and Tables

**Table 1:** Identification of the constituents from extracts of *G. ulmifolia* by LC-DAD-MS/MS.

Peak	RT (min)	Compound	UV (nm)	FM	Negative mode (*m*/*z*)		Positive mode (*m*/*z*)
					MS [M−H]^−^	MS/MS	MS [M+H]^+^
					341.1090	—	—
2	1.2	NI	—	C_6_H_10_O_8_	209.0303	—	—
3	1.2	Quinic acid	—	C_7_H_12_O_6_	191.0571	—	193.0717
4	1.4	Citric acid	—	C_6_H_8_O_7_	191.0198	—	193.0343
5	1.5	Citric acid derivative	—	C_6_H_8_O_7_	191.0195	—	193.0341
6	2.4	NI	—	C_14_H_18_O_9_	329.0882	—	—
7	4.4	NI	—	C_14_H_19_NO_7_	312.1078	—	336.1057^Na^
8	5.8	NI	—	C_11_H_12_N_2_O_2_	203.0814		205.0970
9	8.6	Epigallocatechin^∗^	278	C_15_H_14_O_7_	305.0687	—	—
10	9.1	Catechin^∗^	278	C_15_H_14_O_6_	289.0735	—	—
11	9.2	PCY-PCY	278	C_30_H_26_O_12_	577.1345	289	579.1501
12	9.5	PDE-PCY	280	C_30_H_26_O_13_	593.1324		—
13	10.2	NI	280	C_15_H_18_O_8_	325.0928		—
14	10.7	5-*O*-*E*-Caffeoylquinic acid^∗^	299,325	C_16_H_18_O_9_	353.0894	191	355.1030
15	11.9	NI	—	C_15_H_19_NO_8_	340.1046	—	—
16	12.1	PCY-PCY	280	C_30_H_26_O_12_	577.1357	407,3399,289,245,161	579.1497
17	12.5	Epicatechin^∗^	280	C_15_H_14_O_6_	289.0716	245,221,187,165	291.0880
18	14.6	PCY-PFI	280	C_30_H_26_O_11_	561.1393	289,245,205,179,164	563.1580
19	15.6	NI	280	C_13_H_14_N_2_O_3_	245.0940	—	—
20	16.8	Di-*O*-deoxyhexosyl-hexosyl quercetin	270,355	C_33_H_40_O_20_	755.2035	300,271,255,179	757.2218
21	17.1	Di-*O*-deoxyhexosyl-hexosyl quercetin	270,355	C_33_H_40_O_20_	755.2044	300,271,255,179	757.2193
22	17.8	*O*-Deoxyhexosyl-hexosyl quercetin	265,350	C_27_H_30_O_16_	609.1472	300,271,255,243	611.1628
23	18.1	*O*-Deoxyhexosyl-hexosyl quercetin	265,350	C_27_H_30_O_16_	609.1467	300,271,255	611.1640
24	18.5	*O*-Hexosyl quercetin	270,350	C_21_H_20_O_12_	463.0903	300,271,255,243	465.1036
25	18.6	*O*-Deoxyhexosyl-hexosyl quercetin	265,355	C_27_H_30_O_16_	609.1473	300,271,255,179	611.1624
		Di-*O*-deoxyhexosyl-hexosyl kaempferol		C_33_H_40_O_19_	739.2087	284	741.2210
26	18.6	PCY-PCY	280	C_30_H_26_O_12_	577.1375	289	579.1508
27	18.9	*O*-Hexosyl quercetin	265,355	C_21_H_20_O_12_	463.0893	300	465.1072
28	20.0	*O-*Pentosyl quercetin	265,350	C_20_H_18_O_11_	433.0775	300,271,255,243	435.0918
29	20.2	*O*-Deoxyhexosyl quercetin	265,350	C_21_H_20_O_11_	447.0937	300	449.1091
30	20.6	*O*-Deoxyhexosyl hexosyl luteolin	265,337	C_27_H_30_O_15_	593.1498	284,255,227	595.16699

^∗^Confirmed by authentic standard. NI: nonidentified; PDE: prodelphinidin; PFI: profisetinidin; PCY: procyanidin; RT: retention time; —: non-observed/detected means.

**Table 2:** Antioxidant activity of aqueous extracts from Guazuma ulmifolia stem bark (GUEsb) and leaves (GUEl).

	DPPH scavenging		
	IC_50_ [*μ*g·mL^−1^]	Maximum activity [*μ*g·mL^−1^]	(%)
AA	6.9 ± 1.0	25	96
BHT	21.5 ± 7.3	75	85
GUEsb	25.2 ± 5.1	100	91
GUEl	39.3 ± 8.8	100	84

IC50 and maximum activity of DPPH free radical scavenging of standard antioxidants and the aqueous extracts from *Guazuma ulmifolia* stem bark (GUEsb) and leaves (GUEl).

**Table 3:** Body mass evolution, food and water intake, hematological parameters, biochemical parameters, and relative mass of the organs of female mice treated with single doses of GUEsb.

Parameters	Control	GUEsb	
		2000 mg·kg^−1^	5000 mg·kg^−1^
Evolution body weight (%)	0.00 ± 1.83	−0.40 ± 1.67	−2.20 ± 1.02
Food intake (g·day^−1^)	14.30 ± 1.10	16.42 ± 1.50	17.30 ± 1.41
Water intake (mL^−1^·day)	26.20 ± 2.43	28.00 ± 1.50	28.85 ± 1.64
WBC (10^3^·*μ*L^−1^)	3.84 ± 0.87	2.80 ± 0.90	3.60 ± 0.60
RBC (10^6^·*μ*L^−1^)	10.30 ± 0.30	9.70 ± 0.33	10.00 ± 0.57
HGB (g·dL^−1^)	13.50 ± 0.41	12.84 ± 0.44	13.52 ± 0.60
HCT (%)	53.10 ± 1.73	49.42 ± 1.90	53.20 ± 2.22
MCV (fL)	52.40 ± 1.10	51.02 ± 0.50	53.30 ± 0.50
MCH (pg)	13.30 ± 0.30	13.30 ± 0.20	13.54 ± 0.10
MCHC (g·dL^−1^)	25.40 ± 0.20	26.12 ± 0.31	25.40 ± 0.30
PLT (10^3^·*μ*L^−1^)	968.8 ± 114.9	1204.2 ± 49.1	1049.2 ± 107.9
Neutrophil (10^3^·*μ*L^−1^)	0.53 ± 0.12	0.33 ± 0.14	0.37 ± 0.08
Linfocyte (10^3^·*μ*L^−1^)	3.30 ± 0.80	2.45 ± 0.80	3.20 ± 0.50
AST (U·L^−1^)	63.50 ± 4.80	59.70 ± 1.10	68.40 ± 15.60
ALT (U·L^−1^)	35.70 ± 4.70	30.70 ± 1.91	30.20 ± 3.21
Urea (mg·dL^−1^)	48.52 ± 3.90	52.22 ± 1.84	53.70 ± 2.21
Creatinine (mg·dL^−1^)	0.20 ± 0.01^a^	0.20 ± 0.01^a^	0.30 ± 0.02^b^
CNS (g·100^−1^ of body weight)	0.41 ± 0.06	0.42 ± 0.05	0.55 ± 0.06
Heart (g·100^−1^ of body weight)	0.44 ± 0.03	0.44 ± 0.02	0.44 ± 0.06
Liver (g·100^−1^ of body weight)	4.17 ± 0.13	3.91 ± 0.12	4.17 ± 0.09
Spleen (g·100^−1^ of body weight)	0.30 ± 0.01	0.30 ± 0.01	0.30 ± 0.01
Lung (g·100^−1^ of body weight)	0.55 ± 0.06	0.52 ± 0.06	0.60 ± 0.04
Kidney (g·100^−1^ of body weight)	1.01 ± 0.02	0.94 ± 0.030	1.10 ± 0.02

CNS = central nervous system; WBC = white blood cells; RBC = erythrocytes; HGB = hemoglobin; HCT = hematocrit; MCV = mean corpuscular volume; MCH = mean corpuscular hemoglobin; MCHC = mean corpuscular hemoglobin concentration; PLT = platelet; AST = aspartate aminotransferase; ALT = alanine aminotransferase. Data were expressed as mean ± SEM. GUEsb = aqueous extract of *G. ulmifolia* stem bark. Different superscript letters indicate statistically significant differences.

**Table 4:** Evolution of body mass, food and water consumption, and relative mass of organs of C57Bl/6 mice induced to oxidative stress with DOX.

Parameters	Control	DOX	DOX + GUEsb
Evolution body weight (%)	1.0 ± 0.85^a^	−5.0 ± 1.17^b^	−6.6 ± 3.02^b^
Food intake (g·day^−1^)	37.1 ± 4.74	28.1 ± 4.11	27.8 ± 3.46
Water intake (mL^−1^·day)	25.9 ± 0.86	22.0 ± 1.85	21.9 ± 1.67
Heart (g·100^−1^ of body weight)	0.48 ± 0.02	0.55 ± 0.05	0.47 ± 0.01
Liver (g·100^−1^ of body weight)	4.36 ± 0.13	4.94 ± 0.16	4.87 ± 0.19
Kidney (g·100^−1^ of body weight)	1.08 ± 0.07	1.05 ± 0.02	1.02 ± 0.01

The data are expressed as the mean ± SEM (*n* = 5). Different letters signify statistical differences at *P* < 0.05. GUEsb = aqueous extract from *G. ulmifolia* stem bark.
